# B vitamins intake and cancer risk: a structured narrative review of evidence on riboflavin, pyridoxine, cobalamin and folate

**DOI:** 10.3389/pore.2026.1612380

**Published:** 2026-06-19

**Authors:** Alexander Bertuccioli, Chiara Maria Palazzi, Irene Asia Massaro, Massimiliano Cazzaniga, Ilaria Cavecchia, Maria Rosaria Matera, Valentina Biagioli, Luca Imperatori, Francesco Di Pierro, Annalisa Belli

**Affiliations:** 1 Department of Biomolecular Sciences, University of Urbino Carlo Bo, Urbino, Italy; 2 Microbiota International Clinical Society, Torino, Italy; 3 Scientific and Research Department, Velleja Research, Milan, Italy; 4 Francesca Pirozzi Foundation ETS, Fano, Italy; 5 Department of Medicine and Technological Innovation, University of Insubria, Varese, Italy

**Keywords:** cancer risk, cobalamin, folic acid, pyridoxine, riboflavin

## Abstract

**Background:**

B vitamins play key roles in one-carbon metabolism, DNA synthesis and methylation, and redox regulation. Their potential association with cancer risk remains debated due to heterogeneous findings and methodological limitations across studies.

**Objective:**

This structured narrative review aimed to summarize and clinically contextualize the available evidence on the relationship between the intake of vitamins B2 (riboflavin), B6 (pyridoxine), B12 (cobalamin) and folate (vitamin B9) and the risk of selected cancers.

**Methods:**

A structured literature search was performed in PubMed up to 2 September 2025. Evidence was synthesized narratively, prioritizing meta-analyses (including dose–response meta-analyses) of observational studies, and including randomized controlled trials on B-vitamin supplementation when clinically relevant. Methodological quality and risk of bias were appraised narratively focusing on key domains such as exposure assessment, confounding, selection bias, and outcome assessment.

**Results:**

Most evidence derives from observational studies assessing dietary intake and circulating biomarkers. Riboflavin and vitamin B6 intake were generally associated with a reduced risk of colorectal cancer in several meta-analyses, including dose–response analyses. Vitamin B12 showed inconsistent associations across cancer sites, with concerns regarding confounding by diet and baseline nutritional status. Folate intake and folic acid supplementation showed mixed findings, with some evidence suggesting protective effects in specific populations, while randomized trials raised concerns about potential adverse effects in selected contexts and dosages. Overall, heterogeneity across studies was substantial, partly explained by differences in exposure definitions, baseline folate fortification policies, and co-interventions.

**Conclusion:**

Available evidence suggests potentially protective associations for riboflavin and vitamin B6—particularly for colorectal cancer—whereas findings for vitamin B12 and folate remain inconsistent and context-dependent. Given relevant methodological limitations and potential biases, further well-designed prospective studies and targeted trials are needed to clarify dose, timing and population-specific effects of B vitamins on cancer risk.

## Introduction

B vitamins are a chemically heterogeneous group of essential water-soluble compounds that play a wide variety of roles in the human body [1–4]. The B-vitamin family includes B1 (thiamine), B2 (riboflavin), B3 (niacin), B5 (pantothenic acid), B6 (pyridoxine), B7 (biotin), B9 (folate), and B12 (cobalamin). Since mammals are unable to synthesize these compounds in sufficient amounts, they must be obtained through the diet.

Most B vitamins are mainly derived from plant-based foods, either directly through plant consumption or indirectly through animal-derived products such as dairy, eggs, and meat. Vitamin B12 represents an exception, as it is synthesized by microorganisms and is mainly obtained by humans through animal-derived foods such as liver, fish, eggs, meat, and dairy products. In humans, vitamin B12 can also be synthesized by colonic bacteria; however, this source is not physiologically available for absorption, since cobalamin absorption occurs in the terminal ileum and depends on intrinsic factor-mediated uptake [1, 3–5]. The gut microbiota may also contribute to the production of several B vitamins. For example, as highlighted in a review on natural products used primarily in the management of overweight [6], the ability of some nutraceuticals to promote the growth of beneficial bacteria such as bifidobacteria has been linked to immunomodulatory processes and to the synthesis of B vitamins.

B vitamins play essential roles as coenzymes in enzymatic reactions that are vital to several biological systems. In their active forms, they participate in the formation of biologically active holoenzyme complexes (coenzyme-enzyme) within different cellular processes [1, 2, 4, 7, 8]. Their functions are diverse but closely interconnected, including the maintenance of energy homeostasis, the synthesis of biologically relevant molecules such as neurotransmitters, and the preservation of structural integrity in tissues such as the nervous system and myelin [1–8].

In this context, despite their well-established biological roles, the relationship between B vitamin intake and cancer risk remains controversial and not yet fully clarified. While several observational studies suggest that adequate dietary intake of B vitamins—particularly those involved in one-carbon metabolism such as folate, vitamin B6, and vitamin B12—may be associated with a reduced risk of certain cancers, particularly gastrointestinal cancers, other evidence points toward neutral or even adverse effects depending on dose, timing, and source of intake.

Randomized controlled trials of folic acid supplementation have generally shown no significant protective effect on cancer incidence and, in some cases, have raised concerns about a potential increase in cancer risk or progression, particularly in populations with pre-existing neoplastic lesions or high baseline exposure [9, 10]. Similarly, long-term high-dose supplementation with vitamins B6 and B12 has been associated with an increased risk of lung cancer in specific subgroups, such as male smokers [11].

Moreover, dose–response analyses and site-specific investigations have suggested heterogeneous associations across different cancer types, with some studies reporting positive associations—for example between vitamin B12 intake and esophageal cancer—while others show null or inconsistent findings [12, 13]. These discrepancies may be explained by differences in study design, baseline nutritional status, genetic variability, background fortification policies, and the distinction between dietary intake and pharmacological supplementation.

Overall, current evidence supports a complex and context-dependent relationship between B vitamins and cancer risk, characterized by potential beneficial effects at physiological levels and possible adverse effects at high doses or in specific clinical contexts. These inconsistencies highlight the need for a structured and critical synthesis of the available evidence.

## Controversies over the use of B vitamins in oncological diseases

The role and potential use of B vitamins in oncology is currently quite controversial. In addition to the essential physiological functions performed by these vitamins, various studies provide evidence of both positive and negative effects. For instance, dietary nutrients involved in one-carbon metabolism, such as folate, vitamin B6, vitamin B12, and methionine, may act as potential protective factors against cancer, particularly pancreatic cancer, by supporting DNA methylation, nucleotide synthesis, and DNA replication and repair. Folate, for example, may influence genome stability and gene expression through its essential role in methionine synthesis and its conversion to S-adenosylmethionine (SAM, the universal methyl group donor for DNA methylation), while vitamin B12 acts as a cofactor in this biochemical reaction [14, 15]. Vitamin B6 is also a cofactor for several key enzymes involved in methyl group metabolism [16]. A deficiency in folate and other methyl-group–related nutrients may increase the risk of pancreatic cancer by altering DNA and RNA methylation, disrupting DNA integrity and repair mechanisms, and increasing DNA damage and genetic mutations [17, 18].

Vitamin B1 (thiamine) is also essential for cellular metabolism, as it supports energy production and DNA synthesis. Its deficiency can cause severe neurological and cardiac issues. In cancer cells, thiamine influences glucose metabolism, potentially slowing tumor growth [19]. It is important to note that thiamine deficiency is more commonly observed in patients with diabetes—a condition that already increases the risk of complications. Since cancer patients may also present with thiamine deficiency, diabetic patients with cancer find themselves in a particularly vulnerable condition [20]. Vitamin B2 deficiency can lead to the development of migraines, anemia, and nervous system damage. Riboflavin affects cancer progression by regulating key processes such as redox reactions and oxidative stress levels. It may enhance the effects of antitumor drugs and induce tumor cell death, also due to its anti-inflammatory properties [19]. However, the effects vary depending on the dosage and cancer type, and the literature is not homogeneous in its findings. Lu et al. [20], in their study involving 377 individuals with gastric cancer (GC) and 756 healthy controls, observed that regular riboflavin intake appears to offer protection against the development of GC, particularly in individuals with specific genetic variants of the MTRR gene. A recent retrospective case-control study explored the potential connection between blood riboflavin levels and the onset of colorectal cancer. The results, following thorough statistical analysis and consideration of confounding factors, suggest that riboflavin may play a role in facilitating the development of this disease, with an increased risk proportional to blood riboflavin concentration. In other words, the higher the riboflavin levels, the greater the apparent risk of developing colorectal cancer [21]. Nicotinamide (NAM), a water-soluble form of vitamin B3 (also known as niacin), is a precursor of NAD+, a key molecule in cellular energy metabolism, participating in both redox and non-redox reactions. NAD+ promotes ATP production, reduces oxidative stress, and activates enzymes such as SIRT1 and PARP1, which are involved in cell regulation and DNA repair. NAM, on the other hand, can inhibit their activity through negative feedback mechanisms [22]. Most research on nicotinamide (NAM) has focused on the chemoprevention of non-melanoma skin cancer (NMSC), one of the most common malignancies in Australia, New Zealand, and North America. Preclinical and clinical studies, including the ONTRAC study (Oral Nicotinamide To Reduce Actinic Cancer), have shown that oral NAM treatment is safe, well tolerated, and significantly reduces the incidence of NMSC and actinic keratoses in high-risk patients [23].

The study by Buqué et al. [24] evaluated the efficacy of NAM in a murine model of HR+ breast cancer, mimicking the immunobiological features of human luminal B breast cancer (HER2−, ER+, VIM−). NAM proved effective both in prevention and treatment by reactivating immune control, mainly through CD8^+^ T lymphocytes. Vitamin B5, a precursor of coenzyme A, has also shown immunostimulatory activity in recent studies. In particular, it enhances T cell responses against infections such as malaria and tuberculosis [25]. More advanced research has revealed that vitamin B5 is involved in modulating mitochondrial metabolism in CD8^+^ T cells, contributing to enhanced antitumor function. CoA supplementation can reprogram T cells toward a Tc22 phenotype, boosting oxidative metabolism (OXPHOS), persistence, and IL-2 production.

This results in improved antitumor responses both in murine models and in combination with anti-PD-L1 therapies. Additionally, high plasma levels of pantothenic acid have been associated with increased efficacy of PD-1 blockade in melanoma patients [26]. Vitamins B2, B6, and B9 are essential for DNA and protein synthesis and also play key roles in the immune response. Their deficiency has been shown to impair both cellular and humoral immunity. Several studies support the role of folic acid in enhancing cellular immunity [27]. Mikkelsen et al. [28] evaluated the effects of B2, B6, and B9 supplementation on the proliferation of U937 pro-monocytic cells, derived from a 37-year-old male patient with histiocytic lymphoma. Their results indicate potential dose-dependent anti-proliferative and anti-migratory effects. The underlying mechanisms may include modulation of angiogenesis, changes in cytokine secretion, alteration of PD-L1 expression, oxidative stress, and nitric oxide synthesis. Nevertheless, several studies have reported a possible association between excessive supplementation of certain B vitamins and the progression of oncologic diseases [9, 11, 29, 30]. Furthermore, B vitamins are generally important for the functioning of the nervous system, and their deficiency can contribute to nerve dysfunction and peripheral neuropathy. The latter is a debilitating side effect that affects up to 90% of cancer patients, and supplementation with B vitamins may prove useful in prevention [31]. Specifically, among the consequences of a vitamin B6 deficiency is peripheral neuropathy; however, given its narrow therapeutic index, high doses could also interfere with the effectiveness of the chemotherapy drugs themselves; therefore, it would be necessary to know the actual deficiency in order to then provide supplementation if needed. Vitamin B12, on the other hand, can be used as a safe prevention for peripheral neuropathy induced by, for example, cisplatin [32]. According to a meta-analysis by Sun et al. [12], vitamin B12 supplementation appears as a potential tool for colorectal cancer (CRC) prevention, particularly in those who consume high doses. Their research also highlighted that the association between the risk of CRC and the total amount of vitamin B12 consumed is more significant than the contribution of diet alone. Finally, the administration of vitamin B6 could also represent a protective strategy against pancreatic cancer [33].

## Materials and methods

This work was designed as a structured narrative review aimed at summarizing and clinically contextualizing the available evidence on the association between the intake of vitamins B2 (riboflavin), B6 (pyridoxine), B12 (cobalamin), and folate (vitamin B9) and the risk of selected cancer outcomes. The review focused primarily on gastrointestinal cancers (particularly colorectal cancer), lung cancer, breast cancer, and ovarian cancer; evidence on melanoma was also considered when available.

### Literature search strategy

A literature search was conducted in PubMed up to 2 September 2025. Search terms were defined to retrieve evidence specifically addressing B vitamins intake (dietary intake and/or supplementation) in relation to oncological outcomes. Keywords included combinations of terms related to B vitamins (e.g., vitamin B2, riboflavin, vitamin B6, pyridoxine, vitamin B12, cobalamin, folate, folic acid) and cancer outcomes (e.g., cancer risk, colorectal cancer, gastric cancer, pancreatic cancer, breast cancer, lung cancer, ovarian cancer, melanoma). Additional relevant studies were identified through manual screening of reference lists of eligible publications.

The initial search yielded a total of 512 records. After removal of duplicates and screening of titles and abstracts, 120 articles were retained for full-text assessment. Following full-text evaluation based on the predefined inclusion and exclusion criteria, a total of 45 studies were included in the final narrative synthesis. The study selection process was conducted by three reviewers, and disagreements were resolved through discussion.

**FIGURE 1:**
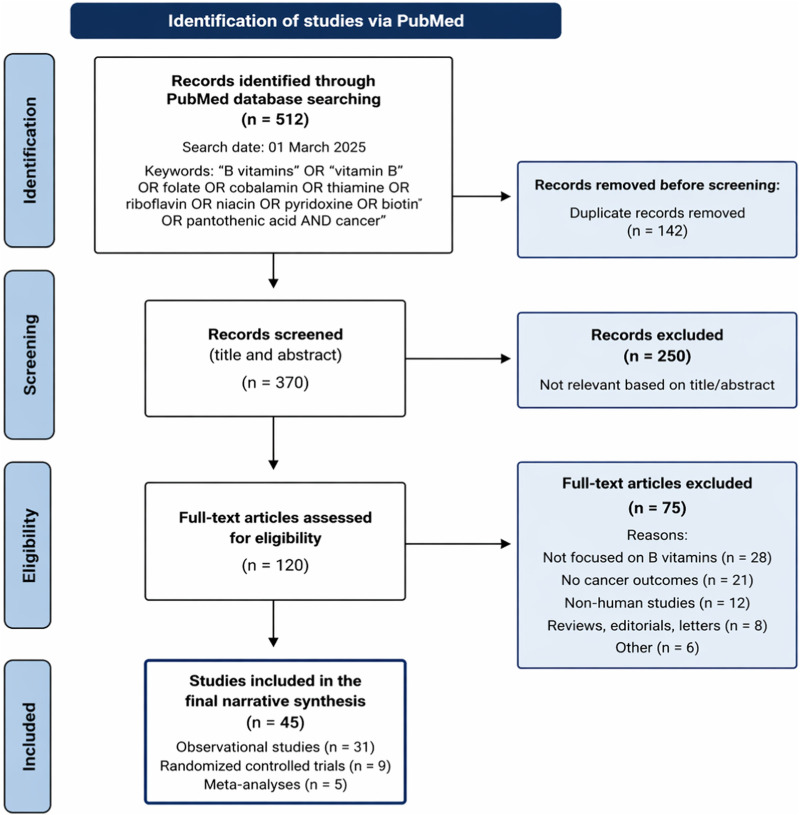
The diagram illustrates the process of identification, screening, eligibility assessment, and inclusion of studies. A total of 512 records were initially identified through database searching. After removal of duplicates, 370 records were screened by title and abstract, of which 250 were excluded. Subsequently, 120 full-text articles were assessed for eligibility, and 75 were excluded based on predefined inclusion criteria. Ultimately, 45 studies were included in the qualitative synthesis.

Although this review was not designed as a systematic review, the study selection process followed a structured and transparent approach inspired by PRISMA recommendations, as showed in the flow-diagram in Figure 1.

### Study selection and inclusion criteria

Evidence was selected according to a structured narrative approach. Priority was given to high-level evidence, in particular meta-analyses and dose–response meta-analyses of observational studies. Randomized controlled trials (RCTs) were also included when they provided clinically relevant information on supplementation effects and safety, with special attention to trials involving folic acid.

Eligible studies were included if they met the following criteria: (i) meta-analyses or pooled analyses of observational studies evaluating B vitamins intake or circulating biomarkers and cancer risk; (ii) RCTs assessing B vitamin supplementation and oncological outcomes (incidence and/or mortality), when available; (iii) studies reporting effect estimates as relative risk (RR), odds ratio (OR), or hazard ratio (HR) with corresponding confidence intervals.

Studies were excluded if they: (i) were not focused on oncological outcomes; (ii) were conducted exclusively in pediatric populations; (iii) investigated nutrients outside the B vitamin group; or (iv) did not provide quantitative effect estimates. Evidence was selected to provide a balanced overview of the available literature, prioritizing meta-analyses, dose–response meta-analyses, prospective cohort studies, and randomized controlled trials when available. Studies were not selected solely on the basis of statistically significant or protective findings, but also included null, inconsistent, or potentially adverse associations in order to reflect the heterogeneity of the evidence.

REV. Three punto 2

Eligibility criteria were defined *a priori* to ensure transparency and reproducibility of the selection process and inclusion/exclusion criteria are summarized in Table 1.

**TABLE 1:** Summary of the inclusion and exclusion criteria and evidence selection strategy applied in the structured narrative review of B-vitamin intake and cancer risk.

Category of evidence	Included	Rationale
Meta-analyses of observational studies	Yes	Considered the highest level of available epidemiological evidence for evaluating associations between B-vitamin exposure and cancer risk
Dose–response meta-analyses	Yes	Included to assess potential quantitative exposure–risk relationships
Prospective cohort studies	Yes	Useful for longitudinal assessment of dietary exposure and cancer incidence
Randomized controlled trials (RCTs) on B-vitamin supplementation	Yes	Included when reporting clinically relevant oncological outcomes, particularly for folic acid supplementation
Studies evaluating dietary intake	Yes	Relevant to habitual B-vitamin exposure in real-world settings
Studies evaluating circulating biomarkers (e.g., PLP, serum B12)	Yes	Included because biomarkers may provide more objective exposure assessment
Studies investigating nutrients other than B vitamins	No	Not relevant to the predefined exposure criteria
Pediatric population studies	No	The review focused on adult populations
Animal studies and *in vitro* studies	No	The review prioritized human clinical and epidemiological evidence
Case reports and narrative opinion papers	No	Considered low-level evidence with limited generalizability

### Narrative risk of bias and methodological considerations

Although this work was designed as a structured narrative review, particular attention was paid to the appraisal of methodological quality and potential sources of bias across the included evidence. In the absence of formal risk-of-bias tools, the assessment was conducted narratively, focusing on bias domains most relevant to nutrition- and oncology-related evidence. Specifically, the following aspects were considered: (i) study design limitations (e.g., retrospective vs. prospective design, observational vs. interventional nature); (ii) selection bias and population representativeness (including eligibility criteria and baseline characteristics); (iii) exposure assessment and classification bias (accuracy of dietary intake and supplementation assessment, dosage reporting, and measurement tools); (iv) confounding and co-intervention effects (including baseline nutritional status, folate fortification policies, comorbidities, concomitant supplementation, and treatment-related variables); (v) outcome assessment bias (definition and measurement of oncological endpoints, follow-up duration, and missing outcome data); and (vi) reporting bias, including selective outcome reporting and publication bias. This narrative appraisal was used to contextualize the strength and reliability of findings and to guide interpretation of inconsistencies among studies, without excluding evidence solely based on methodological limitations.

### Data extraction and narrative synthesis

For each eligible publication, data were extracted on study design, cancer outcome(s), population characteristics, exposure definition (dietary intake, supplementation, circulating levels), effect measures (RR/OR/HR), and the main quantitative findings. Results were organized and summarized by vitamin (B2, B6, B12, folate) and by cancer site. When multiple meta-analyses addressed the same cancer outcome, emphasis was placed on the most comprehensive and methodologically robust evidence. Findings were synthesized narratively, highlighting consistency, heterogeneity, and clinically relevant patterns across the literature.

## Results

Available evidence from meta-analyses and selected randomized controlled trials was narratively summarized to evaluate the association between B-vitamin intake, circulating biomarkers, and supplementation and the risk of solid tumors. The main findings are presented below, organized by vitamin and cancer site. To facilitate interpretation, a summary table of key findings by vitamin and cancer site is provided (Table 2). Results were structured according to both vitamin type and cancer site, in order to facilitate comparison across exposures and oncological outcomes. When more than one meta-analysis or high-level study was available for the same vitamin–cancer association, findings were interpreted together rather than relying on a single estimate.

**TABLE 2:** Summary of associations between B vitamin intake and risk of solid tumors.

Cancer site	Vitamins	Observed association	References	OR or RR
Colorectal cancer	Vitamin B6 (dietary intake)	Protective association (OR for highest vs. lowest intake reported as protective). Stronger effects reported for women and for colon cancer	[11]	RR = 0.56 (0.46–0.67, p < 0.001) [11]
	Vitamin B6 (biomarker: PLP)	Stronger protective association than dietary intake (PLP-based estimate reported as more protective)	[14]	30%–50% risk reduction [14]
	Vitamin B2 (dietary intake)	Protective association reported (OR < 1)	[33]	OR = 0,83 (95% CI: 0,75–0,91) [33]
	Vitamin B9 (dietary folate)	Dietary folate intake is associated with reduced CRC risk	[34]	RR = 0.71 (95% CI: 0.59–0.86) OR = 0.77 (95% CI: 0.62–0.95) [34]
	Folic acid (supplementation)	No effect of folic acid supplementation on CRC risk in trials	[35]	OR = 1.01 (95% CI: 0.82–1.23) [35]
	Vitamin B12 (intake dose-response)	Dose–response analyses suggest a modest inverse association, but overall evidence for CRC remains largely neutral	[30]	Total intake: RR = 0.963; 95% CI 0.928, 0.999; dietary intake: RR = 0.914; 95% CI 0.856, 0.977 [30]
Esophageal cancer	Vitamin B6 (dietary intake; dose-response)	Higher intake is associated with reduced risk	[11, 36]	RR = 0.57, 95% CI: 0.47–0.69, p < 0.001 [11] OR = 0.59, 95%CI: 0.52–0.66) [36]
	Vitamin B9 (dietary intake; dose-response)	Higher intake is associated with reduced risk	[36]	OR = 0.62, 95% CI: 0.56–0.68 [36]
	Vitamin B12 (dietary intake; dose–response)	Positive association specifically for esophageal adenocarcinoma; +2% risk per +1 µg/day reported	[36]	Or: 1.30; 95% CI: 1.05–1.62 [36]
Gastric cancer	Vitamin B6 (dietary intake)	Inverse association for GI cancers overall; evidence for gastric cancer alone remains limited and inconsistent	[11]	RR = 0.66 (95% CI: 0.57–0.76, p < 0.001) [11]
Pancreatic cancer	Vitamin B6 (dietary intake)	Strong inverse association reported for dietary intake	[11]	RR = 0.64, 95% CI: 0.44–0.93, p = 0.02 [11]
	Vitamin B6 (biomarker: PLP; dose–response)	Strong inverse association for PLP; dose–response reported as risk reduction per PLP increment	[14]	RR not reported
	Vitamin B9 (dietary folate)	Case–control suggested inverse association; prospective cohorts did not confirm (overall heterogeneous)	[37]	RR = 1.07, 95%CI: 0.59–1.93, p = 0.78) [37]
	Vitamin B12	No significant association reported	[9, 31, 38]	RR = 0.99; 95% CI: 0.59–1.38 [31]
Breast cancer	Vitamin B9 (intake; dose-response)	No overall association [37]; lowest risk at ∼200–320 µg/day; increased risk above ∼400 µg/day reported [39]. Possible protective effect in high alcohol consumers suggested [39] but not confirmed in prospective cohorts alone	[37, 39, 40]	RR = 0.99; 95% CI, 0.98–1.01; p = 0.361 [37] RR = 0.89, 95% CI: 0.66–1.20, p = 0.30) [40]
Ovarian cancer	Vitamin B9 (dietary folate and total folate)	No significant association for dietary or total folate intake	[41]	RR = 1.06, 95% CI = 0.89–1.27, p = 0.120 [41]
Melanoma	Vitamin B9 (supplementation)	One trial-level meta-analysis suggested reduced risk [35]; larger pooled RCT analyses reported null [37]	[35, 37]	RR = 0.47 (95% CI: 0.23–0.94) [35] RR = 1.04 (95% CI: 0.66–1.64) [37]

Evidence for colorectal cancer was the most consistent across B vitamins. In a meta-analysis of 28 observational studies, Lai et al. [34] reported an inverse association between dietary vitamin B6 (pyridoxine) intake and colorectal cancer risk (OR 0.80; 95% CI 0.68–0.94), while circulating pyridoxal 5′-phosphate levels showed a stronger protective association (OR 0.54; 95% CI 0.35–0.84). Favorable associations were particularly evident among women and for colon cancer specifically. Similarly, an observational meta-analysis by Liu et al. [36] found a protective association between vitamin B2 (riboflavin) intake and colorectal cancer risk (OR 0.83; 95% CI 0.75–0.91).

Findings for vitamin B9 (folate/folic acid) were more heterogeneous. Moazzen et al. [42] reported that randomized controlled trials of folic acid supplementation showed no significant effect on colorectal cancer risk (RR 1.07; 95% CI 0.86–1.43), whereas prospective cohort studies of dietary folate intake indicated a significant risk reduction (RR 0.71; 95% CI 0.59–0.86). Differences in exposure assessment and background folate fortification were suggested as potential contributors to heterogeneity. Heterogeneity and small-study effects were reported when provided by the original meta-analyses, without performing new quantitative syntheses.

For vitamin B12 (cobalamin), a dose–response meta-analysis by Sun et al. [12] indicated a modest inverse association with colorectal adenoma and cancer risk (RR 0.963 per +4.5 μg/day), with stronger effects observed for dietary intake compared with total intake. However, variability across studies and potential confounding by dietary patterns were noted.

Beyond colorectal cancer, a dose–response meta-analysis by Qiang et al. [13] reported that higher dietary intake of vitamin B6 and folate was associated with a reduced risk of esophageal cancer. In contrast, vitamin B12 intake showed a positive association with esophageal adenocarcinoma, with each additional 1 μg/day associated with a 2% increase in risk.

In a large field synopsis including 121 observational studies, Mocellin et al. [16] found that higher dietary intake of vitamin B6 was associated with a reduced risk of gastrointestinal cancers overall (RR 0.68; 95% CI 0.61–0.75), although site-specific findings for gastric cancer were less consistent.

Regarding pancreatic cancer, Wei and Mao [33] observed strong inverse associations for vitamin B6, with relative risks of 0.63 (95% CI 0.48–0.79) for dietary intake and 0.65 (95% CI 0.52–0.79) for circulating pyridoxal 5′-phosphate. Their dose–response analysis indicated a 9% risk reduction per 10 nmol/L increment in pyridoxal 5′-phosphate levels. No significant associations were identified for vitamin B12. For folate, Fu et al. [39] reported a protective association in case–control studies (OR 0.78; 95% CI 0.66–0.92), which was not confirmed in prospective cohorts (RR 0.85; 95% CI 0.69–1.05).

For breast cancer, a dose–response meta-analysis of prospective studies by Zhang et al. [40] found no overall association between folate intake and cancer risk (RR 0.97; 95% CI 0.90–1.05). A J-shaped relationship was observed, with the lowest risk between 200 and 320 μg/day and an increased risk above 400 μg/day. Additional analyses suggested potential protective effects of dietary folate among women with high alcohol consumption, although these associations were not consistently observed when restricted to prospective studies alone [41].

For ovarian cancer, a meta-analysis of observational studies by Wang et al. [35] found no significant association with folate intake, either dietary (RR 0.90; 95% CI 0.77–1.06) or total intake (RR 1.06; 95% CI 0.89–1.27). Regarding melanoma, a meta-analysis of randomized controlled trials by Qin et al. [37] suggested a reduced risk associated with folic acid supplementation (RR 0.47; 95% CI 0.23–0.94). However, larger pooled analyses of randomized trials involving more than 50,000 participants did not confirm this finding and reported null associations [10].

## Discussion

The purpose of this narrative synthesis was not to provide an exhaustive mapping of all available evidence, but rather to offer a clinically oriented interpretation of the most robust and methodologically informative findings on B-vitamin intake, biomarkers, and cancer risk, with particular attention to sources of heterogeneity and potential biases.

Overall, the most consistent evidence suggests an inverse association between vitamin B6 (pyridoxine) and its active biomarker pyridoxal 5′-phosphate and the risk of gastrointestinal cancers, particularly colorectal and pancreatic cancer. This pattern is biologically plausible given the central role of vitamin B6 in one-carbon metabolism, DNA synthesis and repair, epigenetic regulation via methylation pathways, and modulation of inflammatory and oxidative stress responses. Across studies, associations were generally stronger in analyses based on circulating biomarkers than on dietary intake, supporting the relevance of objective exposure assessment and reducing concerns related to measurement error in dietary questionnaires [16, 33, 34].

For vitamin B9 (folate), findings were more context dependent. Dietary folate intake was generally associated with reduced cancer risk in observational studies, whereas randomized controlled trials of folic acid supplementation showed null effects. This divergence suggests that dose, timing of exposure, baseline folate status, and background fortification policies may critically influence observed associations. In addition, differences between naturally occurring folates and synthetic folic acid may partly explain the lack of concordance between observational and interventional evidence. Therefore, observational evidence on dietary folate should not be directly translated into support for folic acid supplementation for cancer prevention. [10, 39, 42].

Evidence regarding vitamin B12 (cobalamin) was inconsistent. While most studies suggested neutral or modest inverse associations with colorectal cancer risk, dose–response meta-analyses indicated a potential positive association with esophageal adenocarcinoma. Importantly, this finding should not be overlooked, as it suggests that cobalamin may have site-specific and context-dependent associations with cancer risk rather than uniformly neutral or protective effects. These heterogeneous findings are consistent with possible effect modification by coexisting factors such as obesity, gastroesophageal reflux disease, alcohol consumption, and overall dietary patterns, as well as residual confounding inherent to observational research [12, 13].

For hormone-related cancers, including breast and ovarian cancer, available evidence remains inconclusive. Overall folate intake was not associated with breast cancer risk, and the presence of a J-shaped dose–response relationship suggests that higher intakes may not confer additional benefit and could be associated with increased risk in certain exposure ranges [40, 41]. No significant associations were observed for ovarian cancer across observational studies [35].

Findings for melanoma were also conflicting. While one meta-analysis of randomized controlled trials suggested a reduced risk associated with folic acid supplementation, larger pooled analyses of randomized trials reported null associations. These discrepancies highlight the influence of study size, duration of follow-up, and underlying population characteristics on observed outcomes [10, 37].

Across several cancer sites, heterogeneity and potential publication bias were reported in some meta-analyses, for example through asymmetry in funnel plots or results from Egger’s test, although these assessments were not uniformly available and no new quantitative evaluations were performed in the present synthesis. Such methodological considerations underscore the need for cautious interpretation of pooled estimates.

Finally, emerging evidence from Mendelian randomization studies provides additional support for a potential protective role of genetically predicted vitamin B6 across multiple cancer sites. While this approach strengthens causal inference by minimizing confounding and reverse causation, findings remain limited in number and require further validation in larger, well-powered analyses [38]. Nevertheless, the potential protective associations observed for vitamin B6 should be balanced against safety considerations. High-dose pyridoxine supplementation has been associated with neurotoxicity, particularly sensory neuropathy, and this risk is clinically relevant when supplementation exceeds physiological requirements or is used long term [43]. Therefore, the available evidence should not be interpreted as supporting high-dose vitamin B6 supplementation for cancer prevention, especially in the absence of documented deficiency.

## Limitations

The present structured narrative review has several limitations, partly related to the observational nature of the available evidence and to heterogeneity in exposure assessment and baseline population characteristics.

First, although the synthesis primarily relied on meta-analyses, a large proportion of the underlying evidence was derived from observational studies, which are inherently susceptible to residual confounding, reverse causation, and dietary misclassification. In many studies, vitamin intake was assessed using food frequency questionnaires, which may lead to under- or overestimation of true exposure. Analyses based on circulating biomarkers, while providing a more objective measure of exposure, were fewer in number and showed substantial methodological heterogeneity across studies.

Second, considerable variability existed with respect to baseline dietary patterns, geographic regions, fortification policies, and genetic backgrounds, all of which may influence B-vitamin metabolism and modify associations with cancer risk. Such heterogeneity likely contributed to the substantial between-study variability reported in several meta-analyses.

Third, the distinction between dietary intake and supplemental sources of B vitamins was not always clearly defined. In particular, randomized controlled trials of folic acid supplementation often used pharmacological doses that may not reflect habitual dietary intake, limiting the direct comparability between observational and interventional evidence.

Finally, small-study effects and publication bias cannot be excluded. Although these issues were assessed and reported in some meta-analyses—for example through funnel plot asymmetry or Egger’s test—such evaluations were not consistently available across all cancer sites and vitamins, and no new quantitative assessments were performed in the present review [44].

## Future prospects

Future research should address these limitations by conducting well-designed prospective cohort studies with repeated dietary and biomarker assessments, integrating genetic and epigenetic markers to clarify mechanisms. Attention should be paid to distinguishing dietary intake, supplementation, and circulating biomarkers, as these exposure categories may reflect different biological and clinical contexts. Randomized trials specifically targeting high-risk subgroups, such as individuals with folate deficiency, high alcohol consumption, pre-existing preneoplastic lesions, or genetic variants affecting one-carbon metabolism, may help to disentangle the role of B vitamins in cancer prevention. Future studies should also clarify dose–response relationships, timing of exposure, baseline nutritional status, and the potential impact of food fortification policies. Moreover, Mendelian randomization approaches should be further expanded to complement traditional epidemiology and reduce the influence of residual confounding and reverse causation. Ultimately, a better understanding of both potentially protective and potentially adverse associations will be crucial to define whether B vitamins can be meaningfully and safely leveraged in cancer prevention strategies.

## Conclusion

This structured narrative synthesis suggests that vitamin B6 (pyridoxine) and its active biomarker pyridoxal 5′-phosphate are inversely associated with the risk of gastrointestinal cancers, particularly colorectal and pancreatic cancer. Dietary folate intake shows signals of potential protective associations in observational settings, whereas folic acid supplementation has not demonstrated benefit in randomized controlled trials. Evidence regarding vitamin B12 (cobalamin) appears largely neutral for colorectal cancer, while dose–response analyses raise the possibility of a positive association with esophageal adenocarcinoma.

For breast and ovarian cancer, available evidence remains inconclusive, and findings for melanoma are inconsistent across study designs and populations. Taken together, the current body of evidence does not support the use of high-dose B-vitamin supplementation for primary cancer prevention in the general population. Rather, these findings are consistent with the importance of achieving adequate B-vitamin intake through balanced dietary patterns, while acknowledging the need for further well-designed prospective studies and targeted trials to clarify dose-, timing-, and population-specific effects.
